# Results of a Randomized Wait-List Controlled Trial of CAYA: A Nurse Case Management HIV Prevention Intervention for Youth Experiencing Homelessness

**DOI:** 10.1007/s10461-024-04544-3

**Published:** 2024-11-12

**Authors:** Diane Santa Maria, Adeline Nyamathi, Marguerita Lightfoot, Mary Paul, Yasmeen Quadri, Nikhil Padhye, Michael Businelle, Higinio Fernandez-Sanchez, Jennifer Torres Jones

**Affiliations:** 1 Cizik School of Nursing, Center for Nursing Research, University of Texas Health Science Center at Houston, Houston, TX USA; 2 Sue & Bill Gross School of Nursing, University of California Irvine, Irvine, CA USA; 3 Oregon Health & Science University-Portland State University School of Public Health, Portland, OR USA; 4 Department of Pediatrics, Baylor College of Medicine, Houston, TX USA; 5 Department of Family and Community Medicine, Baylor College of Medicine, Houston, TX USA; 6 TSET Health Promotion Research Center, University of Oklahoma Health Sciences Center, Oklahoma City, OK USA

**Keywords:** Youth Homelessness, HIV Prevention, PrEP, nPEP, Condom use, Substance use

## Abstract

**Abstract:**

Youth experiencing homelessness (YEH) face challenges that increase their susceptibility to HIV/STIs. Nurse case management is effective in managing the complex needs of populations experiencing homelessness and reducing HIV risk. A randomized wait-list control study conducted between September 2019 to May 2023 evaluated the CAYA “Come As You Are” intervention. This nurse-led HIV prevention for YEH aged 16–25 years focused on the uptake of HIV prevention methods: pre- and post-HIV exposure prophylaxis (PrEP, nPEP), HIV/STI testing and treatment, sober sex, and condom use. Secondarily, we examined intervention impact on housing stability. Descriptive statistics were calculated by study arm. Multiple imputation (m = 10) was used for missing values and intervention effects were estimated from Bayesian multilevel models with noninformative priors. Participants (*N* = 450) were 21.1 years old on average, 62% Black, 11% Hispanic, 11% White, and 10% other race and reported being homeless for an average of 3 years. An intervention effect was found for PrEP use, which showed a larger increase from baseline to first follow-up (OR = 3.27; 95% Cr.I.: 1.13 to 10.14). No intervention impact was found for nPEP use, HIV and STI cases, sober sex, or condom use. Sheltering arrangements improved from baseline to the first follow-up in both groups with increase in shelter stability (OR = 3.85; 95% Cr.I.: 1.61 to 10.30) and decreased shelter transiency (OR = 0.29; 95% Cr.I.: 0.14 to 0.60). This study demonstrates that a personalized, nurse-led HIV prevention approach increased uptake of some but not all HIV prevention strategies among YEH.

**Clinical Trial Registration number:**

NCT03910218.

## Abstract (es)

**Resumen:**

Los jóvenes sin hogar enfrentan desafíos que aumentan su susceptibilidad al VIH/ITSs. Las intervenciones de enfermería ser efectivas para abordar las necesidades complejas de las poblaciones sin hogar y para reducir el riesgo de infección por VIH. Se llevó a cabo un estudio controlado aleatorizado con lista de espera entre septiembre de 2019 a mayo de 2023 para evaluar la intervención CAYA (“Come As You Are”). Esta intervención, dirigida por enfermeras, fue diseñada para jóvenes sin hogar de entre 16 y 25 años y se centra en la adopción de métodos de prevención del VIH, incluyendo Profilaxis Preexposición (PrEP) y Profilaxis Posterior a la Exposición (nPEP), pruebas y tratamiento del VIH/ITS, así como el uso de preservativos y prácticas sexuales seguras. Además, se evaluó el impacto de la intervención en la estabilidad de la vivienda. Se calcularon estadísticas descriptivas para comparar las características demográficas iniciales del grupo. Se utilizó la imputación múltiple (m = 10) para manejar los valores faltantes, y los efectos de la intervención se estimaron mediante modelos multinivel Bayesianos con valores previos no informativos. Los participantes (*N* = 450) tenían una edad promedio de 21,1 años. El 62% eran Negros, el 11% Hispanos, el 11% Blancos y el 10% de otras razas. Los jóvenes reportaron haber estado sin hogar por un promedio de 3 años. La intervención mostró efectividad en el uso de PrEP, con un aumento significativo desde el inicio hasta el primer seguimiento en el grupo de intervención en comparación con el grupo de control (OR = 3,27; IC del 95%: 1,13 a 10,14). Sin embargo, no se observó significancia estadística en el uso de nPEP, en la incidencia de VIH y ETS, en la abstinencia sexual o en el uso de condones. Esto se evidenció por la presencia de efectos temporales en la estabilidad del alojamiento (OR = 3.85; IC del 95%: 1.61 a 10.30) y la reducción de la transitoriedad del alojamiento (OR = 0.29; IC del 95%: 0.14 a 0.60). Este estudio demostró que un enfoque de prevención del VIH personalizado y dirigido por enfermeras aumentó la adopción de algunas, pero no todas, las estrategias de prevención del VIH entre los jóvenes sin hogar.

## Introduction

### HIV Risk in Youth Experiencing Homelessness

Youth experiencing homelessness (YEH) face numerous challenges that may increase their vulnerability to HIV and other sexually transmitted infections (STIs) [1] and YEH have worse health outcomes if they acquire HIV [2]. YEH often engage in behaviors associated with increased risk for HIV infection such as early sexual debut, substance use, trading sex for necessities, and having multiple sexual partners [3, 4]. While this can vary depending on local resources, YEH are often disconnected from healthcare and prevention services [5, 6] and, therefore, have lower knowledge of HIV prevention methods and are less likely to use condoms when compared to housed youth [7, 8]. Further, nearly a quarter of YEH have reported experiencing sexual exploitation [9] and have been sexually assaulted since becoming homeless [9]. Unfortunately, few (< 30%) YEH access healthcare services after being sexually assaulted [10] and therefore, miss the opportunity for access to post-exposure prophylaxis (nPEP) for HIV and STIs.

Screening for HIV and STIs, prevention and care education, and access to pre-exposure prophylaxis (PrEP) and nPEP are effective interventions to help reduce HIV risk. Daily Truvada for HIV PrEP was first approved in the United States in 2012 for individuals facing susceptibility. Despite the efficacy and safety of PrEP, there remains a significant portion of PrEP-eligible people who are not currently receiving it [11]. YEH report not knowing about PrEP despite having elevated risk for HIV [7]. Studies have found that only 20–30% of YEH knew about PrEP and only 1% were currently taking PrEP for HIV prevention [5, 12, 13]. In a large, multi-city study (*n* = 1427) of YEH (58% male, 81% youth of color, 31% LGBTQ+), authors found that 71% had little to no knowledge of PrEP [14]. Furthermore, although over 60% of YEH perceived themselves as vulnerable to HIV, only 14% indicated that they were actively trying to prevent HIV. However, once informed, YEH were interested in PrEP [12, 14]. Similar challenges are present for the uptake of nPEP. For example, in a study of YEH who indicated that they have been sexually assaulted, only 29% of youth sought post-assault healthcare where they could access nPEP [10]. Increased education and access to PrEP and nPEP as part of the comprehensive Ending the HIV Epidemic Initiative [15] is needed for YEH. This needs to be delivered in a format that breaks down access barriers and promotes uptake and retention in care by being co-located with other homeless services, especially with the literature suggesting that 59–63% of YEH were interested in taking PrEP [12] but continue to struggle to access services [16–19].

### Mental Health and Substance Use

Mental health disorders and substance use further increase vulnerability to HIV [20, 21]. Depression, suicide attempts, and posttraumatic stress are high in YEH, with drug overdose and suicide as the leading causes of death among YEH [22]. Mental health disorders can indirectly be a risk factor for HIV by leading to increased substance use, which can be associated with unsafe injection practices and riskier sexual practices [23]. Higher rates of substance use have been shown in YEH relative to those with more stable housing [24] with studies finding between 69 and 86% meeting the criteria for a substance use disorder [25, 26]. Substance use and stress negatively impact HIV risk [13, 27]. Having sex while high or intoxicated on drugs or alcohol can lead to risky sexual behaviors, increased sexual partners, and reduced condom use. Stress predicts condomless sex, inconsistent condom use, more sexual partners, and substance use [28–34], with rates being twice as high in YEH than housed youth [35, 36].

### Barriers to Health Care Access

YEH are highly transient and geographically mobile, making engaging in healthcare challenging. Access to healthcare is a significant obstacle for YEH, as they often lack health insurance and face discrimination and intersecting stigmas (e.g., racial/ethnic, age, gender identity, housing status, socio-economic) from healthcare professionals and systems [37]. Only about one-third of street-dwelling youth and half of sheltered youth utilize healthcare regularly [38]. YEH who use substances or have mental health issues experience extreme barriers to accessing care [35, 39, 40] with only one-third who need care accessing it [39, 41]. The lack of access to healthcare increases the risk of poor health outcomes, including HIV and STI infections [6, 40]. The most common barriers include transportation, lack of health insurance, and healthcare costs [40]. Therefore, HIV prevention interventions that meet youth where they are currently and that provide comprehensive case management may increase reach, reduce costs, and improve care engagement.

Social and structural determinants of uptake and adherence to HIV prevention strategies are well documented and include insurance coverage, costs, stigma, transportation, and healthcare utilization [42]. Yet, many YEH struggle to access HIV prevention information and services despite their heightened vulnerability. Interventions to address HIV prevention among YEH are sorely needed, but research in this area is limited. The importance of addressing mental health and substance use disorders in a population vulnerable to HIV is crucial in developing these interventions. Despite HIV prevention initiatives for YEH and the federal efforts to Ending the HIV Epidemic in the U.S., HIV rates remain disproportionately high and PrEP/nPEP rollout efforts have not successfully reached YEH. Interventions need to be co-located with other homeless youth services to promote ease of access to programs [43] while also addressing mental health, substance use, and housing needs often faced by YEH.

### Motivational Interviewing Based Nurse Case Management

YEH often distrust authority [33, 34], hampering access to healthcare and HIV prevention. Thus, to effectively scale-up HIV prevention for YEH, interventions must be delivered by trusted, PrEP-competent providers who offer comprehensive healthcare and help navigate YEH to substance use treatment and mental healthcare when needed [44–46]. Nurse case management has proven effective in managing the complex needs of populations experiencing homelessness and HIV risk reduction [47, 48]. A nurse case manager can help facilitate the integration of education across various HIV prevention strategies and provide health and social service navigation. By providing HIV and STI screening, PrEP and nPEP education and access, and care coordination in one visit, a nurse case manager can provide YEH with a single linkage point for comprehensive HIV prevention.

Case management that utilizes motivational interviewing (a counseling style that helps evoke motivation for change) [49], which has been shown to help youth with behavioral change related to substance use and safer sexual practices [50, 51], can help meet the complex needs of YEH in a way that promotes shared decision-making and motivation for prevention behavior adoption. Motivational interviewing-enhanced nurse case management provides a structure for the nurse and the individual to develop goals for HIV prevention by utilizing available resources and driving improved motivation to achieve health outcomes. Behavioral feedback can also enhance motivation for behavior change among youth. Utilizing behavioral feedback technology via smartphones is an acceptable and accessible way to provide behavioral feedback to this population [52, 53].

Nurse case management integrated with motivational interviewing and behavioral feedback can assist with shared decision-making strategies. Interventions should also be co-located and delivered in partnership with housing and shelter providers [54–56]. YEH want access to healthcare, sexual health counseling, text-based supportive messaging, and individualized supportive services to support PrEP uptake [14, 19, 57, 58]. PrEP awareness and uptake is also improved by providing youth-friendly, technology-assisted behavioral goal setting, nurse case management, and access to healthcare [59].

### Purpose

This study aimed to evaluate the impact of the CAYA intervention on the adoption of HIV prevention methods, including PrEP, nPEP, HIV and STI testing and treatment, sober sex, and condom use. This nurse-led intervention provided comprehensive HIV prevention education for YEH aged 16–25 years. Group differences were assessed between the intervention group and the wait-list control group. We sought to identify differences in the impact on HIV prevention strategies, mental health, and substance use among YEH.

### Methods Design

A randomized wait-list controlled trial was conducted with two groups to assess the efficacy of the intervention compared to the standard of care. One group received the intervention while the other group served as a wait-list control. The study focused on measuring the adoption of HIV prevention methods, including PrEP, nPEP, HIV and STI testing and treatment, sober sex, and condom use. Secondary outcomes evaluated the intervention’s impact on mental well-being, substance use, and housing stability. Efficacy measures were assessed three months post-intervention. The complete study protocol is published elsewhere [60].

### Study Setting and Recruitment

Participants were recruited from various locations serving YEH in the greater Houston, TX and Harris County area including drop-in centers, shelters, clinics, and through street outreach. Recruitment strategies included group-based study introduction sessions, distribution of flyers and recruitment letters, online advertisements, and leveraging relationships with associated organizations and networks. Study staff were present at shelters, drop-in centers, and during outreach events 3–5 times a week to recruit and accept referrals from staff. To enhance representativeness, a sample was recruited with diverse gender and sexual orientation.

Prior to and after the COVID-19 pandemic-related shutdowns, recruitment and enrollment were conducted in-person at YEH-serving organizations. The widespread local shutdowns resulted in an eight-month pause in face-to-face recruitment which led to a shift to virtual recruitment. When face-to-face recruitment resumed, the enrollment delays caused by the pandemic were addressed by adding recruitment sites, encouraging participant referrals with incentives, enhancing online social media recruitment methods, and initiating virtual recruitment.

Participants received a $10 gift card if they referred another YEH that enrolled in the study. Recruitment flyers were posted on social media with options for interested individuals to send direct messages, calls, texts, or emails. Virtual recruitment entailed youth screening and consenting over the phone for youth who contacted the team based on a referral from a friend or after encountering a social media or poster study ad. If eligible, individuals completed the baseline survey sent via text or email and set up one in-person visit at the university to complete enrollment. Transportation was provided for participants to get to the university via a HIPAA-compliant rideshare service and staff members followed protocols for COVID-19 screening and transmission prevention. Once COVD-19 restrictions were lifted, study staff returned to in-person recruitment but continued allowing virtual recruitment.

Study staff were trained to follow study procedures for recruitment, as well as trauma-informed approaches, crisis management, and ethical practices in research practices. Study staff approached the youth to describe the study, screen for eligibility, and obtain informed consent in a quiet area. Potential participants were informed at each encounter that participation would not affect their ability to receive housing, mental health, or healthcare services. Study staff maintained a weekly presence at the recruitment sites throughout the study to facilitate both recruitment and follow-up efforts.

### Inclusion and Exclusion Criteria

Individuals aged 16 to 25 years who were experiencing homelessness or unstable housing and had no plans to leave the Houston metropolitan area during the study were included. Experiencing homelessness was defined broadly using the McKinny-Vento Homeless Assistance Act definition encompassing various living situations such as sleeping on the streets, in shelters, hotels, with friends or family, or in temporary rented accommodation where you are unsure where you will be able to stay in the next 30 days. Individuals with very low literacy, assessed during study screening, were excluded, and those experiencing acute mental distress or suspected intoxication were encouraged to return to be screened for enrollment at a later time (see CONSORT diagram).

### Ethical Considerations

This research was reviewed and approved by the Institutional Review Board at the University of Texas Health Science Center (Protocol # HSC-SN-18-0993). Every participant provided written consent prior to engaging in this study and was reminded consistently throughout the study that participation was completely voluntary. For YEH who were minors, written consent was obtained, and parental consent was waived due to their unaccompanied state.

### Intervention Description

The CAYA intervention was based on the Comprehensive Health Seeking and Coping Framework [61]. It consisted of six face-to-face sessions with a nurse and a behavioral assessment and feedback app [62]. Motivational interviewing techniques and shared decision-making strategies were used to assess the participants’ current situation, develop care plans, and mutually set HIV prevention goals. The intervention focused on promoting HIV prevention behaviors, including PrEP/nPEP use, HIV/STI testing, avoiding sex while using substances, and condom use. HIV testing and PrEP eligibility assessments were performed and access to PrEP was facilitated through a PrEP navigator. While the nurse sessions were originally designed to be face-to-face, COVID-19 pandemic restrictions required a virtual option to continue the study during the local shutdown, thus resulting in a hybrid delivery format based on participant preferences and access to public meeting areas and shelters. Approximately 60% of all delivered intervention sessions were conducted in-person. The study-based app delivered brief daily behavioral assessments asking about HIV prevention behaviors and allowed participants to track their goal progression in real time on the study-issued phone. A comprehensive description of the intervention is provided in another published protocol paper [60].

### Enrollment

Participants were randomly assigned to either the intervention or wait-list control group using a computer-generated blocked 2:1 allocation process. The intervention group included 303 participants, while the wait-list control group included 147 participants for a total of 450 YEH. All enrolled participants were provided a smartphone with a basic data plan to use for the duration of the study to stay in contact with the study team and complete the daily behavioral assessments that informed the behavioral interface (intervention group only). The study definition of enrolled included participants who completed the consent and baseline survey, received their randomization assignment and smartphone. This stepwise enrollment process was utilized to preserve study resources due to the transiency of the population.

### Measurements

Demographics such as age, gender identity, race/ethnicity, sexual orientation, and current housing [63] (last night stay) were collected in the baseline survey. The baseline survey also assessed behaviors including number of sex partners in the last three months [64], history of juvenile justice and foster care involvement, and length of the current episode of homelessness.

Baseline and follow-up surveys collected self-report PrEP/nPEP use, condom use (*How often in the past three months did you use a condom during sexual intercourse?* and *The last time you had sexual intercourse*,* did you or your partner use a condom?*), sober sex (*Did you drink alcohol or use drugs the last time you had sex?*) [64], and substance use in the last 30 days [65, 66]. Rapid tests were administered by study staff to assess presence of HIV (INSTI HIV-1/HIV-2 Antibody Test [67]) and Syphilis (Syphilis Health Check [68]) antibodies and commercially available urine testing kits were used to test for Gonorrhea and Chlamydia [69]. Treatment for HIV, any STI, and nPEP uptake (after a sexual assault) was also collected in baseline and follow-up surveys. For HIV treatment among those who indicated that they were HIV positive, participants were asked whether they were currently taking medication to treat HIV. If a participant reported that they had an STI, they were asked if they had been treated for that STI. For nPEP uptake, they were asked if they had taken the medication after a sexual assault.

Mental health issues were assessed using a single item (*Are you currently experiencing problems with your mental health?*). The Kessler Psychological Distress Scale (K6) was used to assess symptoms of distress [70] with a score equal to or greater than 13 indicating a probability of a serious mental illness, a score under 13 indicating that a serious mental illness is unlikely, and a score of 8 to 12 indicating moderate distress. The Patient Health Questionnaire (PHQ9) [71] was used to assess the severity of depression [72]. Housing stability was measured using this question, “*Where did you stay last night (examples provided)?*”. Housing transiency was measured by asking, “*During the past three months*,* have you spent the night in any of the following places (examples provided)*?”. Housing uncertainty was assessed by inquiring, “*How many separate occasions have you been unsure of where you will sleep at night in the last three months*?” [73].

### Outcomes

The primary aim of the study was to determine whether the CAYA intervention increases uptake of HIV prevention strategies including PrEP and nPEP uptake, HIV and STI testing and treatment, sober sex, and condom use when compared with youth in the wait-list control group (*N* = 450; aged 16–25 years) at immediate post-intervention. An exploratory aim was to determine whether CAYA improved mental health, substance use, and housing stability when compared with wait-list control youth at immediate post-intervention.

### Statistical Analysis

Distributions of demographic variables were compared at baseline to assess whether randomized recruitment with 2:1 ratio in the CAYA and control groups had resulted in even distribution of age, gender identity, race/ethnicity, sexual orientation, type of last night sheltering, length of homelessness, history of juvenile justice or foster care involvement, mental health issues, and substance use at baseline. The chi-squared test and Welch two sample t-test were used for inference among categorical and continuous variables, respectively. The nonparametric Wilcoxon rank sum test was used when continuous variables had skewed distributions. Treatment effects were assessed from the interaction term of the intervention group and time in longitudinal models that also included the main effects of group and time. For this purpose, Bayesian multilevel models were calculated with the *brms* [74–76] package (version 2.20.3) in *R* that resulted in the models being fitted in *Stan* [77, 78] (version 2.34) after translation to the *Stan* programming language and compiled in C + + before being returned to *brms* for post-processing. The advantages of this method included the use of Hamiltonian sampling that allowed for greater flexibility in the choice of priors. Flat priors were used for the population-level parameters and Student’s t distribution for the standard deviations and cluster-level distributions, or random effects. Outcomes belonged to the family of normal distributions (Kessler and PHQ-9 scales for mental health), or cumulative categorical distribution for an ordinal scale (condom use), or Bernoulli distribution (PrEP/nPEP use, condom use at last encounter, sober sex, HIV/STI cases, substance use, and mental health issues). The intervention effect was considered statistically significant if the 95% credible interval for the posterior distribution of the parameter corresponding to the interaction term of intervention group and time excluded zero, which is equivalent to excluding the null value, 1, for odds ratios of a categorical outcome.

While missing fractions were low at baseline for many outcomes, loss to follow-up resulted in larger missing fractions at the immediate post-intervention time point. There was no reason to believe that missing values would be informative. Multiple imputation was used as a safeguard against the possibility of biased results from inclusion of only the complete cases in the models. The *mitml* [79] package (version 0.4.5) was used to interface with *jomo* [80] (version 2.7.6), which allowed specifying multilevel imputation models for both continuous and categorical variables. The imputation model was identically structured to the analysis model to ensure alignment of the methods. Imputed data sets (*m* = 10) were analyzed with the Bayesian multilevel modeling approach described above. The *brms* package has the built-in ability to summarize the posterior distributions of parameters resulting from models fitted to multiple data sets. For conditional outcomes (sober sex, condom use, condom use at last encounter) that were asked only if the participant had indicated having sex, the number of missing values were estimated by imputing any missing values of the condition (having sex), which resulted in an estimate of the available pool of participants who could have responded to the conditional questions. Lastly, for exploratory outcomes of interest that were only available for small subsets (< 30) of participants, the distributions were compared with Fisher’s exact tests at baseline and at the immediate post-intervention follow-up. The analysis was implemented in *R* [81] (version 4.3.2) in the *RStudio* [82] environment (version 2023.12.1 + 402).

## Results

### Participant Enrollment and Demographics

**Fig. 1:**
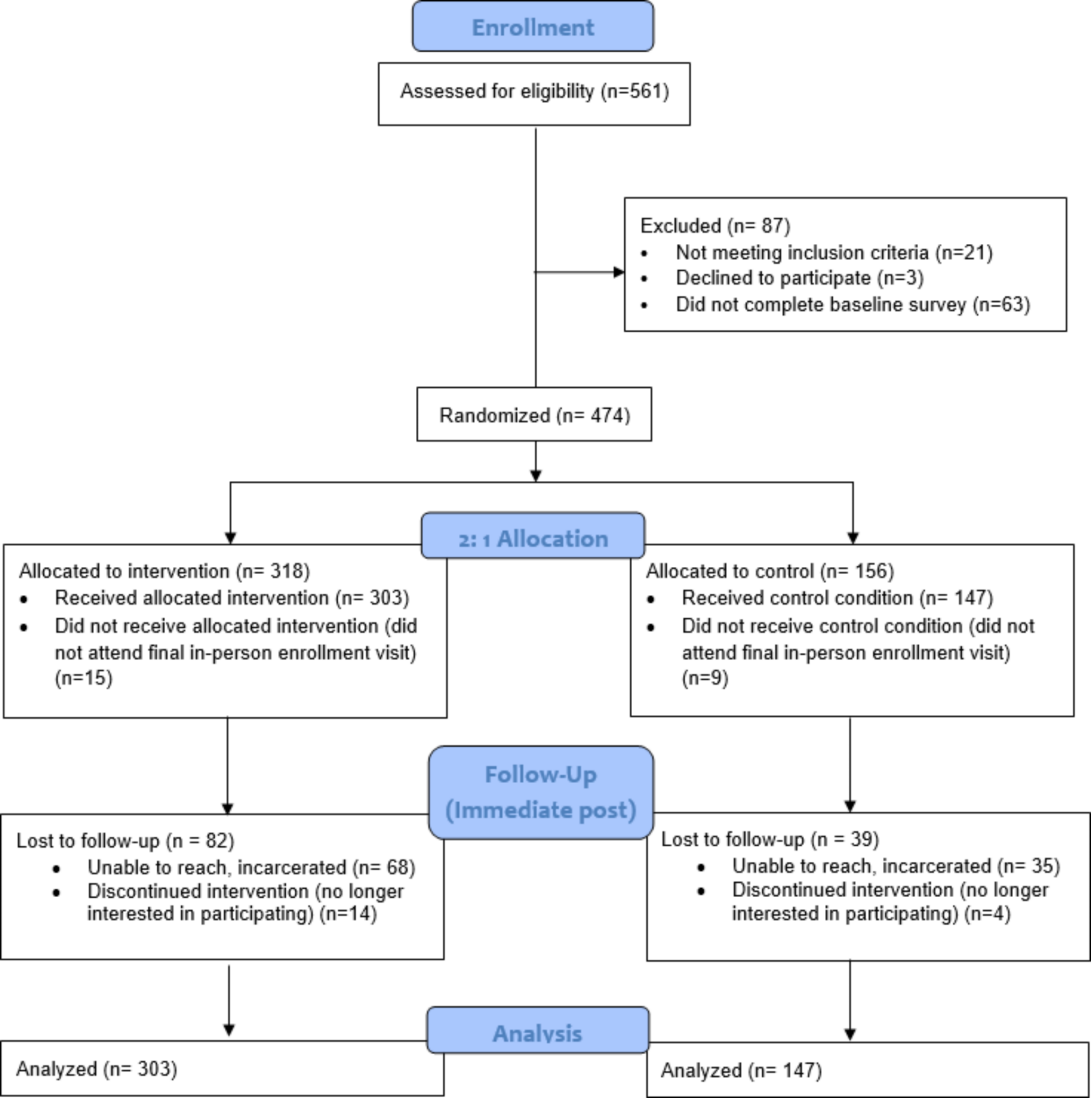
CAYA CONSORT flow diagram

As Fig. 1 indicates, 450 participants were enrolled with a 2:1 intervention to control group randomization (intervention = 303; control = 147; see CONSORT). Most participants (64%) were recruited from temporary emergency shelters, followed by drop-in centers (24%), and virtual recruitment, including youth referrals and responses to flyers and social media posts (12%). The latter recruitment strategies were implemented due to pandemic-related restrictions in access to shelters and service providers. On average, YEH completed five out of six possible sessions with the nurse completed. The follow-up rate at the immediate post-intervention time period was 73.1% (329/450) with no difference by group assignment.

The average age of participants was 21.1 years (SD = 2.1), gender distribution was 50.2% cisgender men, 43.3% cisgender women, 6.4% other gender, and the racial/ethnic distribution was 62.0% Black, 16.0% Hispanic, 11.3% White, and 10.7% other races (included Asian, Native American, Native Hawaiian or Pacific Islander). The length of homelessness had a skewed distribution with a median value of 3.0 years. A history of juvenile justice involvement was reported by 33.1% of the sample while 33.3% reported having been in foster care. Mental health issues were reported by 37.2% of participants at baseline, while the mean (SD) values were 9.3 (6.2) on the Kessler-6 scale indicating moderate distress and 8.7 (7.1) on the PHQ-9 scale indicating mild depression. Refer to Table 1 for a detailed description of the sample characteristics. There were no statistically significant demographic differences between the intervention and wait-list control groups. For the outcomes, missing values at baseline ranged from 0.002% for mental health issues to 13.6% for Gonorrhea and Chlamydia tests due to the challenges faced with pandemic-related shutdowns and the addition of virtual recruitment. Missing value fractions at the immediate post-intervention follow-up ranged from 27% for shelter stability to 47% for Gonorrhea and Chlamydia tests largely due to loss of access to participants during the pandemic. The median values of missing fractions were 2.8% at baseline and 30.4% at the 3-month follow-up. Apart from the longitudinal increases in missing fractions, associations were not found between missingness and demographic or other baseline characteristics.

**Table 1:** Sample description at baseline

Characteristic	Overall, *N* = 450^*1*^	Control, *N* = 147^*1*^	NCM, *N* = 303^*1*^	*p*-value^2^
Age	21.1 (2.1)	21.3 (2.1)	21.0 (2.1)	0.109
Gender				0.228
Female	195 (44%)	57 (39%)	138 (46%)	
Male	226 (50%)	77 (53%)	149 (49%)	
Other	27 (6.0%)	12 (8.2%)	15 (5.0%)	
Race/Ethnicity				0.530
Black	279 (62%)	94 (64%)	185 (61%)	
Hispanic	72 (16%)	25 (17%)	47 (16%)	
Other	46 (10%)	15 (10%)	31 (10%)	
White	51 (11%)	12 (8.2%)	39 (13%)	
Sexual Orientation				0.954
Heterosexual	286 (64%)	95 (65%)	191 (63%)	
Bisexual	89 (20%)	28 (19%)	61 (20%)	
Gay	27 (6.0%)	10 (6.8%)	17 (5.6%)	
Lesbian	21 (4.7%)	6 (4.1%)	15 (5.0%)	
Pansexual	13 (2.9%)	3 (2.0%)	10 (3.3%)	
Other	14 (3.1%)	5 (3.4%)	9 (3.0%)	
Last Night’s Shelter				0.563
Youth shelter	207 (46%)	70 (48%)	137 (45%)	
Friend/Family home	70 (16%)	16 (11%)	54 (18%)	
Adult shelter	69 (15%)	25 (17%)	44 (15%)	
Other	56 (12%)	19 (13%)	37 (12%)	
Street, park, or outside	27 (6.0%)	10 (6.8%)	17 (5.6%)	
My own place	20 (4.5%)	7 (4.8%)	13 (4.3%)	
Ever Had Sex	313 (71%)	92 (66%)	221 (74%)	0.086
Had Sex within 3-mo	213 (68%)	67 (73%)	146 (67%)	0.286
Sex Partners in 3-mo				0.298
Mean (SD)	2.0 (1.6)	2.1 (1.5)	2.0 (1.6)	
Median (IQR)	1 (1, 3)	1 (1, 3)	1 (1, 2)	
History of Juvenile Justice	149 (33%)	53 (37%)	96 (32%)	0.340
History of Foster Care	150 (33%)	48 (33%)	102 (34%)	0.850
Length of Homelessness				0.850
Mean (SD), *year*	3.7 (3.7)	3.9 (4.3)	3.6 (3.4)	
Median (IQR), *year*	3 (1, 5)	3 (1, 5)	3 (1, 5)	
Mental Health Issues	167 (37%)	53 (36%)	114 (38%)	0.867
Substance Use	248 (55%)	79 (54%)	169 (56%)	0.677

^*1*^ Mean (SD); *n* (%)

^*2*^ Welch Two Sample t-test; Pearson’s Chi-squared test; Wilcoxon rank sum test

### Prevalence of HIV and STIs, Sober Sex, and Condom Use

Among the entire sample at baseline, 21.3% of participants tested positive for any STI including HIV and 4.0% tested positive for more than one STI. Specifically, 3.5% were HIV-positive, 6.0% had Syphilis, 4.6% had Gonorrhea, and 11.6% tested positive for Chlamydia. The average missing fraction at follow-up for the HIV and STI tests was 46.1% (range: 45.1–46.9%) while the average missing fraction at follow-up was 29.1% (range: 26.9–31.1%) for all other outcomes of interest. Past 30-day substance use was high with 55.4% indicating use. Similarly, 69.6% indicated that they were high the last time they had sex. Overall, condom use was low for those who indicated that they were sexually active with only 18.4% indicating that they always used a condom in the last three months and only 30.4% reported that they used a condom during their last sex.

### Intervention Effects

Analysis of multiple imputed data sets indicated that there was an intervention effect on PrEP uptake, which showed a larger increase from baseline to first follow-up in the intervention group compared to the control group (OR = 3.27; 95% Cr.I.: 1.13 to 10.14). The result was of similar magnitude when missing values were ignored and the model was fit to the raw data (OR = 3.60; 95% Cr.I.: 1.23 to 11.59), where *Cr.I.* denotes the Bayesian credible interval. The 95% credible interval has the straightforward interpretation that there is 95% probability that the true estimate would lie within the specified interval [83]. Sheltering arrangements also improved from baseline to the first follow-up in both groups, as indicated by statistically significant time effects for increase in shelter stability (OR = 3.85; 95% Cr.I.: 1.61 to 10.30), decreased shelter transiency (OR = 0.29; 95% Cr.I.: 0.14 to 0.60), and decreased shelter uncertainty (OR = 0.41; 95% Cr.I.: 0.25 to 0.67). Treatment effects were not found for the shelter measures, condom use measured on an ordinal scale, condom use at last encounter, sober sex, substance use, HIV, and STI incidence (see Table 2). Although each of three measures of mental health showed stronger improvement in the intervention group compared with the control group, the intervention effects on mental health outcomes were not statistically significant.

**Table 2:** Intervention effects

Characteristic	Control, *n* = 147		CAYA, *n* = 303			95% Cr. I.	
	Baseline^1^	Follow-up^1^	Baseline^1^	Follow-up^1^	OR/β^2^	Lower	Upper
PrEP Use	18 (12%)	15 (14%)	20 (6.7%)	37 (17%)	3.27	1.13	10.14
Consistency of Condom Use^*3*^	2.3 (1.5)	2.6 (1.7)	2.6 (1.6)	2.8 (1.5)	0.83	0.27	2.62
Condom at Last Sex^*4*^	31 (34%)	28 (44%)	84 (39%)	56 (42%)	0.63	0.19	2.04
Sober Sex^*4*^	31 (34%)	17 (27%)	64 (29%)	36 (27%)	1.37	0.43	4.53
HIV Positive	9 (6.6%)	4 (5.3%)	6 (2.1%)	6 (3.5%)	9.71	0.46	592.5
Any Bacterial STI (Syph/Gon/Chlam)	20 (14%)	7 (8.6%)	56 (19%)	23 (13%)	0.80	0.22	2.70
Syphilis Positive	6 (4.3%)	4 (5.2%)	20 (6.9%)	12 (7.1%)	0.54	0.07	4.28
Gonorrhea Positive	5 (3.9%)	0 (0%)	13 (5.0%)	3 (1.8%)	1.05	0.11	13.59
Chlamydia Positive	12 (9.4%)	3 (4.1%)	33 (13%)	11 (6.6%)	1.13	0.20	6.00
Past 30-Day Substance Use	79 (54%)	49 (46%)	169 (56%)	114 (53%)	1.57	0.69	3.67
Mental Health Issues	53 (36%)	34 (31%)	114 (38%)	63 (29%)	0.76	0.35	1.66
Kessler-6 Scale	9.0 (6.5)	8.5 (6.4)	9.5 (6.1)	8.3 (6.0)	-0.86 (β)	-2.14	0.36
PHQ-9 Scale	8.6 (7.2)	8.6 (8.2)	8.8 (7.0)	7.7 (6.7)	-1.11 (β)	-2.72	0.48
Shelter Stability	12 (8.2%)	22 (20%)	24 (7.9%)	43 (19%)	1.08	0.37	3.05
Shelter Transiency	63 (43%)	28 (27%)	135 (45%)	47 (22%)	0.60	0.25	1.40
Shelter Uncertainty	4 (2, 9)	3 (1, 5)	5 (2, 10)	2 (1, 5)	0.72	0.39	1.30

^*1*^ n (%); Mean (SD); Median (IQR); descriptive statistics are based on non-missing values

^*2*^ Odds Ratio or β for intervention effect measured by the interaction between time and treatment group. Estimates are after multiple imputation of missing data

^*3*^ Item conditional upon having sex in the last 3 months; *n* = 70 in Control group and *n* = 148 in CAYA group

^*4*^ Item conditional upon having ever had sex; *n* = 96 in Control group and *n* = 223 in CAYA group

### STI Treatment and nPEP Uptake

Treatment rates were assessed among those who tested positive for HIV or an STI and those who complied with uptake of nPEP or 30 days after a sexual assault. While there was no significant difference in treatment rates for HIV or STIs, treatment uptake increased in both groups (HIV = 78–86%, STIs = 76–94%) suggesting that participating in this type of study may have improved use of available resources. At baseline, 27.4% of youth (*n* = 122) indicated that they had been sexually assaulted, and of those, 35.2% (*n* = 43) sought care. Among 20 participants who were prescribed nPEP, 65.0% (*n* = 13) complied with nPEP uptake. At the immediate post-intervention follow-up three months later, 22.5% (*n* = 72) indicated that they had been sexually assaulted and of those, 41.7% (*n* = 30) sought care. The proportion of those who complied with nPEP was nearly unchanged at 63.6% (*n* = 7). The frequencies of treatment for HIV or STIs and compliance with nPEP uptake were insufficient for longitudinal modeling; inference with Fisher’s exact tests at baseline and at the immediate post-intervention follow-up did not indicate differential proportions in the control and intervention groups (see Table 3).

**Table 3:** Exploratory outcomes

Characteristic	Stage	Overall	Control^1^	CAYA^1^	*p*-value^2^
Treated for STI	Baseline	22 (76%)	7 (78%)	15 (75%)	> 0.999
		*N* = 29	*N* = 9	*N* = 20	
	Follow-up	28 (93%)	7 (88%)	21 (95%)	0.469
		*N* = 30	*N* = 8	*N* = 22	
Treated for HIV	Baseline	7 (78%)	4 (80%)	3 (75%)	> 0.999
		*N* = 9	*N* = 5	*N* = 4	
	Follow-up	6 (86%)	3 (100%)	3 (75%)	> 0.999
		*N* = 7	*N* = 3	*N* = 4	
nPEP Compliance	Baseline	13 (65%)	3 (50%)	10 (71%)	0.613
		*N* = 20	*N* = 6	*N* = 14	
	Follow-up	7 (64%)	3 (75%)	4 (57%)	> 0.999
		*N* = 11	*N* = 4	*N* = 7	

^*1*^ *n* (%); *N*

^*2*^ Fisher’s exact test

## Discussion

This study describes critical evidence of the efficacy of a community-based, nurse-led, HIV prevention intervention (CAYA) among a large sample of youth who represent the demographic diversity frequently found among YEH. Findings suggest that the CAYA intervention increased PrEP uptake among a large sample of YEH who were recruited from shelters and through community outreach. While this increase in PrEP uptake was modest when considering the proportion of participants eligible for PrEP based on their risk level, it is promising given the dearth of interventions found to have any positive impact on PrEP uptake among this subgroup of youth [2]. Given the high prevalence of HIV among this population found in this study (3.5%) and with Harris County being one of the 48 counties highlighted in the Ending the HIV Epidemic, this continues to be a subgroup of youth who demonstrate continued need. While PrEP uptake is a positive outcome, this study did not focus on PrEP adherence. Therefore, additional research is needed among YEH to determine theory-driven and acceptable ways to promote medication adherence and test those interventions for efficacy.

Despite condom use being one of the goals that youth could focus on in their effort to curtail their HIV risk, CAYA had no significant impact on increasing condom use with low rates (34–39% at last sex) across the entire sample. Similarly, there was no improvement in rates of sober sex, decrease in substance use, or HIV and STI results. While we explored the uptake of nPEP post-sexual assault, there were insufficient numbers to assess an intervention effect. That said, due to the high number of sexual assaults reported by youth in the sample, it continues to be an area of HIV prevention that needs further research and intervention development.

There were high rates of HIV and STIs at baseline and the immediate post-intervention follow-up for the entire sample. Among the entire sample, 21% had an STI when tested for HIV, Syphilis, Gonorrhea, and Chlamydia. Further, with 6% of the sample testing positive for Syphilis, less than 40% using condoms at last sex, and high rates of unplanned pregnancy among YEH [84], this data suggests a critical and acute need to increase testing and treatment among this population to prevent the devastating impact of congenital syphilis. Given the high dependence on emergency department (ED) visits for access to any healthcare services, especially in states such as Texas without expanded Medicaid, the ED may be a key site to implement opt-out testing for all pregnant persons [85].

While housing was not the main focus of this study, sheltering needs were addressed at each case management visit with the nurse given the connection between stable housing and reduced HIV risk [86]. We found that youth in both study arms saw marked improvements in their housing stability, reduced transiency, and reduced uncertainty in their housing needs further supporting the need for HIV prevention interventions that utilize a case management strategy that addresses the social determinants of health as part of HIV prevention. Mental health outcomes did not improve due to the intervention. Further, there were high levels of need among the population with over 1/3 (37.2%) indicating that they had mental health needs. Psychological distress was high among the sample, as well as depressive symptoms suggesting that YEH continue to face challenges accessing adequate mental health services.

The challenges that arose during the COVID-19 pandemic severely impacted the trial. Recruitment started nine months before the shutdown experienced in March, 2020. While it is impossible to determine the full extent of the impact on recruitment, intervention delivery, and retention, challenges were numerous and described elsewhere [87]. Other limitations that should be considered when analyzing the findings include the sample of youth being recruited from one large urban area in the South. Therefore, findings may not be generalizable to other regions or rural areas. That said, the sample does represent a very racial/ethnic and gender diverse group of youth who approximate the homeless population in this urban area. While most of the youth (64%) were recruited from temporary shelters, having representation from nearly 40% of non-shelter connected youth is a notable achievement. However, it is hard to determine how many youth with the highest levels of disconnection from services may still be missing from this study.

HIV and STI testing was intended to supplement the self-report in the surveys, however, it was critically interrupted by the COVID-19 pandemic reductions in access to shelters and other services agencies which resulted in more missing data than was anticipated. While the study faced anticipated and unanticipated challenges, there were key strategies that the study used to enhance future research with this population. Maintaining flexibility, cultivating strong relationships with community partners, having YEH participate in all phases of the study through the Youth Working Group, utilizing technology and social media to promote retention, and fostering a diverse research team that represents the heterogeneity of the YEH served were critical lessons learned [87]. These strategies fostered high levels of intervention engagement with the vast majority of participants attending five of the six possible sessions with the nurse.

### Policy Implications

Efforts to address the disproportionate burden of HIV and other STIs among YEH must prioritize interventions grounded in principles of health equity. Tailored strategies should be implemented, and the systemic inequities and structural barriers faced by this population should be acknowledged and addressed [56, 88, 89]. For example, E-WORTH is an impactful intervention specifically designed for Black women. It addresses mechanisms for HIV/STI prevention while also considering the structural racism that Black women encounter [90]. We recommend, like others [91], prioritizing comprehensive sexual health education within current programs, particularly focusing on condom use, regular STI testing and treatment, and ensuring equitable access to pre- and post-exposure prophylaxis (PrEP). These initiatives must be culturally competent and affirming of diverse identities and experiences within the YEH community. Given the prevalence of trauma and sexual violence among YEH, trauma-informed care approaches must be integrated into HIV prevention efforts to improve health outcomes [92, 93]. Providing accessible post-sexual assault support services is essential to mitigate trauma-related barriers to care and promote holistic well-being among YEH as well as promote engagement in post-assault care where nPEP and doxy-PEP can be accessed within a timeframe that promotes efficacy.

## Conclusion

CAYA shows promising evidence of increasing HIV prevention behaviors among YEH, particularly, increasing access to and uptake of PrEP. Housing stability also increased in both groups, suggesting that participation in research may have collateral benefits for YEH by connecting them to social resources. Future work must build off this positive momentum by using implementation science strategies to examine the effectiveness and the ability to replicate these outcomes with other YEH communities with a multi-site study design that involves multiple regions and takes place in real-world settings among resource-limited homelessness provider systems. Further, working with youth to co-design and test PrEP medication adherence interventions that can support PrEP uptake and adherence is also critical. Long-acting injectable PrEP may offer additional advantages for YEH if barriers to accessing the medication and testing are attenuated. Next steps entail exploring the sustainability of the gains found among YEH who participated in CAYA.
